# Systemic and local cytokine profile and risk factors for persistent allergic airway inflammation in patients sensitised to house dust mite allergens

**DOI:** 10.1186/s12890-021-01798-8

**Published:** 2021-12-21

**Authors:** Laura Tamasauskiene, Brigita Sitkauskiene

**Affiliations:** 1 Department of Immunology and Allergology, Lithuanian University of Health Sciences, Eiveniu str. 2, Kaunas, Lithuania; 2 Laboratory of Immunology, Department of Immunology and Allergology, Lithuanian University of Health Sciences, Eiveniu str. 2, 50009 Kaunas, Lithuania

**Keywords:** Allergy, Asthma, Rhinitis, Persistent allergic airway diseases, House dust mite, Cytokines

## Abstract

**Objective:**

To evaluate cytokine profile, vitamin D status, symptom score and quality of life in patients with persistent allergic airway diseases sensitised to house dust mites (HDM) in comparison with healthy individuals.

**Material and methods:**

Patients sensitized to HDM with persistent AR and having symptoms for at least 2 years with or without AA were involved into the study. Measurements of vitamin D level in serum and IL-10, IL-13, IL-17, IL-22, IL-33 and IFN-gamma in serum and nasal lavage were performed by ELISA.

**Results:**

Eighty-one subjects were involved into the study. Serum IL-10 concentration was higher in patients with AR than in patients with AR and AA (6.71 ± 1.73 vs. 1.98 ± 0.24, *p* < 0.05). IFN-gamma level in nasal lavage was higher in patients with AR and AA than in patients with AR (*p* < 0.01) and healthy individuals (*p* < 0.05) (7.50 ± 0.37 vs. 6.80 ± 0.99 vs. 6.50 ± 0.22). Serum IL-22 negatively correlated with IL-22 in nasal lavage, whereas serum IFN-gamma positively correlated with IFN-gamma in nasal lavage. Positive correlation between serum IL-17 and total IgE and negative correlation between IL-17 in nasal lavage and eosinophils in nasal smear were found in patients with AR and AA. Serum IFN-gamma decreased the risk of AR for healthy individuals. Serum IL-10 and vitamin D decreased risk for development of AA for patients with AR. IL-22 in serum and IL-10 and IL-33 in nasal lavage increased this risk.

**Conclusion:**

Novel cytokines such as IL-22, IL-17 and IL-33 and vitamin D may be involved in pathogenesis of persistent airway inflammation in patients sensitized to HDM.

## Introduction

The prevalence of allergic airway diseases—allergic rhinitis (AR) and allergic asthma (AA)—increases despite modern methods of diagnosis and treatment [1–3]. These chronic diseases that usually affect children and young adults is highly associated with poorer quality of life, disturbed social life, daily activity and increased leave day at school and work [4, 5]. Rhinitis and asthma are heterogenous diseases and manifest in different phenotypes which may depend on endotypes [2, 6]. One of the most frequent phenotype of these diseases is allergic [7, 8]. Moreover, AR and AA are very often diagnosed together, that is why the hypothesis of united airway disease was proposed [9, 10].

Despite growing knowledge of pathogenesis of AR and AA and discovery of biomarkers and even modern individualised therapies, the diagnosis and treatment of these diseases are challenging. In addition, most of the disease endotyping and phenotyping research is conducted in severe forms of allergic diseases nowadays, and studies with mild and moderate forms are lacking [11]. Moreover, it is important to investigate local and systemic inflammatory markers because they may not always reflect to each other. For example, Vincente et al. investigated patients with sleep apnoea, which is associated with pharyngeal inflammation, and found differences in inflammatory markers between pharyngeal lavage and plasma [12]. Similarly, it is known a new phenotype of AR without systemic IgE-sensitisation tested by skin prick test and serum allergen-specific IgE called local AR (LAR) [13]. In this situation the main target of investigation should be local biomarkers.

Ordinarily, the main role in pathogenesis of allergic airway diseases is given to T lymphocyte helper (Th) 1 and Th2 ratio imbalance and enhanced production of interleukin (IL) 4, IL-5 and IL-13 by Th2 cells [2, 6, 14]. However, there is an increasing evidence that other subtypes of T lymphocytes, such as Th17 and Th22, may be involved in the pathogenesis of allergic airway diseases.

Th17 are characterized by the production of IL-17, but also may secrete other cytokines such as TNF-α, IL-22, IL-26 and IFN-γ [15]. IL-17 is known to be expressed not only by Th17 but also by other adaptive and immune cell types, including T cells, natural killer (NK) cells, and innate lymphoid cells [16]. IL-17 family cytokines bind to the receptor IL-17R which is expressed on non-immune cells including epithelial cells, airway smooth muscle cells and fibroblastic cells [17]. There are evidences that IL-17 can increase level of pro-inflammatory cytokine and neutrophil count [18]. Moreover, there is a theory about possible interaction between IL-17 and Th2 produced cytokines and total immunoglobulin (Ig) E level and IL-17 link with increased survival of eosinophils [19–21].

Another novel subtype of T lymphocytes is Th22, which is identified by the production of IL-22 [22]. Now it is known that IL-22 can be secreted by Th1, Th2, Th17, Th22, natural killers, and innate lymphoid cells [23]. IL-22 is a member of the IL-10 family of cytokines and plays its role via a heterodimeric transmembrane receptor complex consisting of IL-22R1 and IL-10R2 and subsequent Janus kinase-signal transducers and activators of transcription (JAK-STAT) signaling pathways [24]. The exact role of IL-22 in allergic airway diseases is not known and scientists provide controversial opinions. Some researchers suggest that IL-22 may increase level of proinflammatory cytokines such as IL-13, IL-5 and IL-33 and is related to the level of eosinophil count and total IgE [25, 26]. However, researchers provide evidence of anti-inflammatory properties of this cytokine which is explained by decreased level of proinflammatory cytokines, eosinophil count and mucus producing cells and increased level of IL-10 [25–27].

There are a lot of factors which are suspected to be important for the risk of development and severity of allergic diseases such as increased urbanization, time spent indoors, antibiotic usage, dietary changes, allergen exposure, air pollution, microbiota changes, and etc. [28]. There is some evidence that low vitamin D level is associated with increased risk and more severe forms of allergic diseases [29, 30]. It is suggested that vitamin D may suppress the production of IgE and mast cell activation, may increase IL-10 production, and may reduce level of proinflammatory cytokines [11, 31–33].

The aim of this study was to evaluate cytokine profile, vitamin D status, symptom score and quality of life in patients with persistent allergic airway diseases sensitised to house dust mites (HDM) in comparison with healthy individuals. We have chosen patients sensitised to HDM, because this allergen is one of the major cause of respiratory allergy and of perennial asthma worldwide [34].

This study is relevant because it investigated novel cytokines such as IL-17 and IL-22 which role in allergic airway inflammation are still not know. Moreover, majority of performed research is experimental with animal models and there is lack of clinical studies where cytokine profile is investigated in systemic (serum) and local (nasal lavage) compartments. This is crucial for understanding the role of cytokines in immune response for perceiving differences between local and systemic inflammation.

## Material and methods

### Study population

Patients with persistent AR diagnosed according to the guidelines of Allergic Rhinitis and its Impact on Asthma (ARIA) and having symptoms for at least 2 years with or without AA diagnosed according to the guidelines of Global Initiative for Asthma (GINA) were involved into the study. The inclusion criteria were hypersensitivity to HDM diagnosed by skin prick test or/and allergen specific immunoglobulin (Ig) E test, no use of local or systemic glucocorticoids or other immunosuppressant drugs for at least 1 month before the study, no use of antihistamines for 1 week before the study and no respiratory infection for at least 1 month before the study. Exclusion criteria were relevant hypersensitivity to other inhaled aeroallergens, malignant diseases and systemic autoimmune or other diseases and treatment with allergen immunotherapy. Healthy patients were involved into the study as the control group.

Physical examination was performed, and demographic data was obtained from all subjects.

Subjects were additionally divided into three groups: (1) AR, (2) AR and AA and (3) control group.

The study was approved by the Kaunas Regional Biomedical Research Ethics Committee (No. BE-2-28). Subjects gave their written informed consent.

### Questionnaires

Patients with AR were asked to complete Total nasal symptom score (TNSS) [35] and Rhinoconjunctivitis Quality of Life Questionnaire (RQLQ) [36].

Patients with AA additionally were asked to complete Asthma control test (ACT) [37] and Asthma Quality of Life Questionnaire (AQLQ) [38].

All subjects had to complete Pittsburgh Sleep Quality Index (PSQI) [39] and Structured Interview Guide for the Hamilton Anxiety Scale (SIGH-A) [40].

The permission to use validated questionnaires (Lithuanian versions) was received from authors.

### Evaluation of allergic sensitization

Allergic sensitization was determined by skin prick test or allergen specific IgE test.

Skin prick test was performed according to the standard protocol with standard inhalant allergens (Diater, Spain) on the inner forearm. Drop of different allergen solution was placed at 3 cm distant from each other. Histamine solution 10 mg/ml was used as a positive control and diluent was used as a negative control. The skin was pricked through the drop using the tip of a lancet (separate lancet was used for all allergen drops). Skin reaction was assessed after 15 min. Wheal was measured using ruler. The test was assumed as a ‘positive’ if diameter of the wheal was at least 3 mm.

Measurement of allergen specific IgE was performed using standard immunoblot analysis according to the manufacturer's instructions (Euroimmun, Germany). Total IgE in serum was measured using enzyme immunoassays (AIA-FAC IgEII Tosoh Bioscience, Japan).

### Lung function measurement

Lung function for subjects with AA was evaluated using pneumotachometric spirometer (Smart PFT, Gmbh, Germany). All subjects were asked to avoid the use of short-acting *β*2-agonists for at least 8 h before the testing. Patients were investigated in a sitting position, and a nose clip was used. Before performing the forced expiration, tidal breaths had to be taken first, then a deep breath taken followed by a further quick, full inspiration. Forced expiratory volume in 1 s (FEV1), forced vital capacity (FVC) and FEV1/FVC ratio were measured. The best value of the three measurements was selected.

### Peripheral blood collection and processing

Peripheral vein puncture was performed for all subjects. Blood samples were drawn into KEDTA tubes for investigation for complete blood count and into serum tubes. Serum tubes were stored at room temperature for 30–60 min. and centrifuged at 3500 rpm for 10 min, and serum was separated and frozen at − 80 °C for further analysis.

### Nasal smear and nasal lavage specimen collection and processing

Nasal smears of patients were obtained by gently swabbing the nasal inferior turbinate with a cotton-tipped swab. The sample was then placed on a surface of glass microscope slide and stained with Giemsa stain for eosinophil detection. All specimens were examined by qualified pathologist.

Nasal lavage fluid was collected for all subjects using 5 ml isotone saline per nostril with reclined neck (about 30 °C from the horizontal) and closed soft palate. After 30 s the subject flexed the neck draining lavage fluid into a sterile vessel. Nasal lavage fluid was frozen at − 80 °C for further analysis (cytokine measurement).

### Laboratory analysis of cytokines and vitamin D and cytokines

Measurements of IL-10, IL-13, IL-17, IL-22, IL-33 and IFN-gamma in serum and nasal lavage were performed by ELISA using commercial kits (Elabscience Biotechnology Inc., USA). Limit of detection was 7.81 pg/ml.

Blood for analysis of vitamin D level was collected during the period of September–March. Subjects who stopped using vitamin D supplements at least three months before the study were involved. 25(OH)D level was measured using Immunoblot.

### Statistical methods

Statistical analysis was performed using statistical program SPSS 20. Non-parametric statistical methods were applied.

Mann–Whitney U and Kruskal–Wallis H tests were applied for comparison of variables between patients with allergic airway diseases and healthy individuals and between patients with AR only, patients with AR and AA and healthy individuals.

Methods of correlation (Spearman’s coefficient) was used to find associations between cytokines, eosinophil count, neutrophil count, lymphocyte count, total IgE, vitamin D, TNSS, ACT, AQLQ, RQLQ, PSQI and SIGH-A.

Logistic regression was applied to find risk factors for development of allergic airway diseases.

A *P* value of < 0.05 was considered statistically significant.

## Results

### Characteristics of studied subjects, lung function and quality of life

Eighty-one subjects were involved into the study. Demographic characteristics are presented in Table 1. Subjects’ distribution according to the age and sex did not differ between the groups. Body mass index (BMI) did not differ between the groups. Duration of nasal symptoms was slightly longer in patients with AR and AA compared with patients with AR only.

**Table 1:** Demographic characteristics, lung function and responses to questionnaires of study population

	Patients with allergic airway diseases (N = 63)		Control group (n = 18)
	Patients with AR (N = 42)	Patients with AR and AA (N = 21)	
Male/female, N	17/25	8/13	3/15
Age, years, mean ± SEM	30.12 ± 1.50	33.43 ± 2.10	34.06 ± 2.85
BMI, kg/m2	24.92 ± 1.03	25.95 ± 1.06	24.53 ± 0.81
Duration of rhinitis symptoms, years, mean ± SEM	10.71 ± 1.60	14.46 ± 2.27	N/A
Duration of asthma symptoms, years, mean ± SEM,	N/A	11.33 ± 2.80	N/A
FEV1, l, mean ± SEM	3.76 ± 0.14	3.50 ± 0.22	3.57 ± 0.16
FVC, l, mean ± SEM	4.34 ± 0.17	3.84 ± 0.22	4.12 ± 0.18
FEV1/FVC, %, l, mean ± SEM	84.40 ± 0.92	83.56 ± 1.33	82.85 ± 1.32
FEV1, % of predicted, mean ± SEM	98.58 ± 1.12	100.25 ± 2.27	106.00 ± 3.30
FVC, % of predicted, mean ± SEM	96.85 ± 1.29	97.00 ± 2.04	105.21 ± 3.44
TNSS, mean ± SEM	4.55 ± 0.36**	3.67 ± 0.50**	0.67 ± 0.30
ACT, mean ± SEM	N/A	19.19 ± 0.93	N/A
RQLQ, mean ± SEM	1.79 ± 0.19	1.38 ± 0.30	N/A
RQLQ domain—sleep	1.67 ± 0.30	1.62 ± 0.42	N/A
RQLQ domain—other symptoms	1.47 ± 0.22	1.40 ± 0.39	N/A
RQLQ domain—practical problems	2.22 ± 0.30^#^	1.25 ± 0.36	N/A
RQLQ domain—nasal symptoms	2.18 ± 0.26	1.61 ± 0.35	N/A
RQLQ domain—ocular symptoms	1.27 ± 0.22	0.92 ± 0.21	N/A
RQLQ domain—activity	2.06 ± 0.20^#^	1.31 ± 0.18	N/A
RQLQ domain—emotions	1.94 ± 0.23	1.39 ± 0.43	N/A
AQLQ, mean ± SEM	N/A	5.51 ± 0.28	N/A
PSQI, mean ± SEM	7.35 ± 0.57	7.75 ± 1.17	5.69 ± 0.52
PSQI component 1	1.22 ± 0.12	1.25 ± 0.14	0.88 ± 0.09
PSQI component 2	1.19 ± 0.15	1.31 ± 0.27	1.13 ± 0.22
PSQI component 3	0.78 ± 0.12	1.00 ± 0.24	1.00 ± 0.18
PSQI component 4	0.68 ± 0.14*	0.75 ± 0.27*	0.13 ± 0.09
PSQI component 5	1.46 ± 0.11	1.50 ± 0.16	1.13 ± 0.22
PSQI component 6	0.30 ± 0.14	0.50 ± 0.27	0.13 ± 0.13
PSQI component 7	1.73 ± 0.14	1.50 ± 0.30	1.31 ± 0.18
SIGH-A, mean ± SEM	10.86 ± 1.45	8.69 ± 2.80	9.19 ± 1.40

^#^*p* < 0.05 compared with healthy individuals

^**^p < 0.01 compared with healthy individuals

FVC in percentage was significantly higher in healthy individuals than in all patients with allergic airway diseases (105.21 ± 3.44% vs. 96.90 ± 1.08%, *p* < 0.05). The tendency of higher FEV1 in percentage in healthy individuals than in patients with allergic airway diseases was also observed (*p* = 0.08). However, lung function parameters did not differ between three studied groups (Table 1).

TNSS score was higher in patients with allergic airway diseases than in healthy individuals (Table 1). There were no significant differences of TNSS, RQLQ, PSQI and SIGH-A scores between patients with AR and AA and patients with AR only (Table 1). However, RQLQ domains assessing practical problems and activity was significantly higher in patients with AR only than in patients with AR and AA. PSQI score was significantly higher in all patients with allergic airway diseases than in healthy individuals (7.47 ± 0.53 vs. 5.69 ± 0.52, *p* < 0.05). Moreover, PSQI component 4 (which describes sleep efficiency) was significantly higher in patients with AR and AR and AA than in healthy individuals. SIGH-A did not differ between patients with allergic airway diseases and healthy individuals.

### Leukocytes, eosinophils, and neutrophils in peripheral blood and nasal smear, IgE and vitamin D

Leukocyte level did not differ between studied groups. Eosinophils count in peripheral blood and serum IgE were significantly higher in patients with allergic airway diseases in comparison with healthy individuals (Table 2). However, eosinophils count in nasal smear and serum vitamin D level did not differ between studied groups. Neutrophils level in percentage was significantly lower in patients with allergic airway disease and AR only than in healthy individuals (Table 2). A tendency was observed that neutrophils in percentage was lower in patients with AR and AA than in healthy individuals (*p* = 0.06). A tendency that neutrophils count was lower in all patients with allergic airway diseases (*p* = 0.12) were observed.

**Table 2:** Leukocytes, eosinophils, and neutrophils count in peripheral blood and nasal smear, serum total IgE and vitamin D level in studied groups

	Patients with AR (N = 42)	Patients with AR and AA (N = 21)	Control group (n = 18)
Leukocytes, × 10/9/l	5.90 ± 0.24	6.57 ± 0.40	6.35 ± 0.32
Eosinophils, × 10/9/l	0.22 ± 0.02	0.31 ± 0.05**	0.13 ± 0.03
Eosinophils, %	3.77 ± 0.38*	4.40 ± 0.70*	2.13 ± 0.42
Neutrophils, × 10/9/l	3.31 ± 0.18	3.72 ± 0.26	3.88 ± 0.24
Neutrophils, %	55.43 ± 1.29*	56.11 ± 1.10	60.74 ± 1.81
Eosinophils in nasal smear, %	11.43 ± 3.21	14.79 ± 5.90	3.94 ± 1.83
Total IgE, kU/l	313.34 ± 64.48**	294.40 ± 60.32**	40.47 ± 11.91
Vitamin D, IU/ml	51.33 ± 3.53	45.85 ± 4.94	49.19 ± 3.05

^*^p < 0.05 compared with healthy individuals

^**^p < 0.01 compared with healthy individuals

Data provided as mean ± SEM

### Cytokines level in serum and nasal lavage

**Fig. 1:**
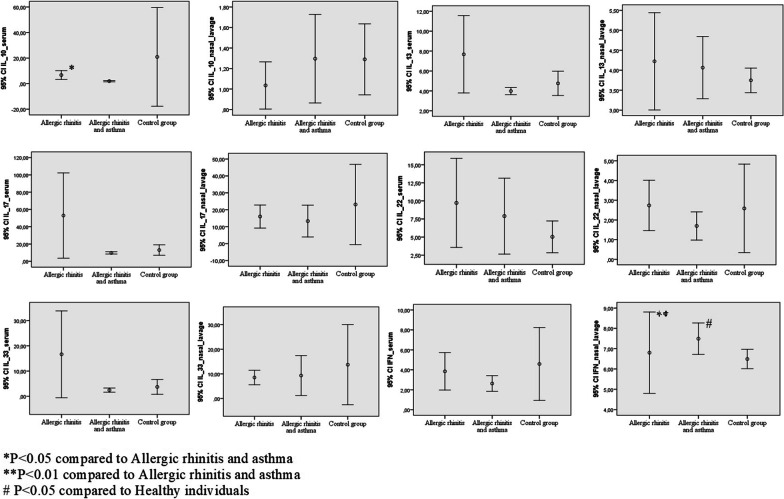
Cytokine level in serum and nasal lavage in patients with AR, AA and AR and healthy individuals

IL-10 concentration in serum was significantly higher in patients with AR only than in patients with AR and AA (6.71 ± 1.73 vs. 1.98 ± 0.24, *p* < 0.05). IFN-gamma level in nasal lavage was higher in patients with AR and AA than in patients with AR only (*p* < 0.01) and in healthy individuals (*p* < 0.05) (7.50 ± 0.37 vs. 6.80 ± 0.99 vs. 6.50 ± 0.22, respectively). All cytokine level in serum and nasal lavage is illustrated in Fig. 1.

### Relation between inflammatory markers, lung function, symptoms, and quality of life

Serum IL-13 negatively correlated with RQLQ in patients with AR and AA. We observed positive correlation between serum IL-17 and total IgE in patients with AR and AA. IL-17 in nasal lavage had negative link with eosinophils in nasal smear. IFN-gamma in nasal lavage had negative correlation with TNSS and RQLQ scores in patients with AR only. IL-33 positively correlated with the count of leukocytes and neutrophils in patients with AR and AA.

Serum IL-22 positively correlated with FEV1 in patients with AR, whereas serum IL-10 positively correlated with FEV1 in patients with AR only and patients with AR and AA. Serum IL-22 negatively correlated with PSQI score, whereas IL-22 in nasal lavage positively correlated with PSQI score in patients with AR and AA. All correlations are presented in Table 3.

**Table 3:** Correlation between cytokines, vitamin D level, scores of symptoms, lung function, quality of life and other inflammatory markers (Spearman’s coefficient)

	IL-22		IL-17		IL-33		IL-10		IL-13		IFN-gamma	
	Serum	Nasal lavage	Serum	Nasal lavage	Serum	Nasal lavage	Serum	Nasal lavage	Serum	Nasal lavage	Serum	Nasal lavage
*Patients with AR*												
TNSS	− 0.19	− 0.04	− 0.19	0.03	− 0.06	− 0.05	− 0.23	− 0.15	− 0.17	0.16	− 0.05	− 0.35*
ACT	N/A	N/A	N/A	N/A	N/A	N/A	N/A	N/A	N/A	N/A	N/A	N/A
RQLQ	− 0.11	0.04	− 0.12	0.10	− 0.02	0.08	− 0.04	− 0.22	− 0.22	0.17	0.04	− 0.37*
AQLQ	N/A	N/A	N/A	N/A	N/A	N/A	N/A	N/A	N/A	N/A	N/A	N/A
PSQI	0.06	0.04	− 0.01	0.22	0.06	0.18	0.20	− 0.02	0.05	0.23	− 0.01	− 0.33
SIGH-A	− 0.15	− 0.20	− 0.10	0.11	− 0.00	0.02	− 0.10	− 0.29	− 0.05	0.03	0.20	− 0.31
Leukocytes, × 10/9/l	− 0.22	− 0.30	− 0.27	− 0.17	− 0.04	0.08	0.01	− 0.13	− 0.25	0.02	− 0.03	0.09
Eosinophils, × 10/9/l	− 0.14	0.09	0.03	0.01	0.02	− 0.02	0.02	− 0.13	0.02	0.00	− 0.05	− 0.10
Eosinophils, %	− 0.14	0.17	0.06	− 0.06	− 0.02	− 0.06	− 0.02	− 0.16	0.06	− 0.03	− 0.14	− 0.14
Neutrophils, × 10/9/l	− 0.06	− 0.14	− 0.26	0.18	− 0.05	0.09	− 0.06	− 0.17	− 0.28	0.06	− 0.07	0.01
Neutrophils, %	− 0.08	0.20	− 0.16	0.23	− 0.02	0.15	− 0.08	− 0.17	− 0.16	0.24	− 0.07	− 0.12
Eosinophils in nasal smear, %	− 0.14	0.24	− 0.03	0.07	0.03	− 0.01	− 0.13	− 0.05	0.05	− 0.12	− 0.15	− 0.20
Total IgE, kU/l	0.02	− 0.13	0.30	0.20	0.30	0.24	0.36*	− 0.03	0.30	0.18	0.33	0.45
Vitamin D, IU/ml	0.015	0.17	0.05	− 0.10	0.02	− 0.02	− 0.10	− 0.01	0.12	− 0.02	0.11	0.15
FEV1, l, mean ± SEM	0.42*	− 0.06	0.31	0.18	0.10	0.13	0.40*	0.12	0.08	0.17	0.30	0.05
*Patients with AR and AA*												
TNSS	− 0.16	0.02	− 0.01	− 0.09	− 0.35	− 0.17	− 0.40	0.31	− 0.31	− 0.10	0.08	0.26
ACT	0.20	0.26	− 0.22	0.37	0.25	0.44	0.12	− 0.04	0.36	0.25	0.31	0.34
RQLQ	− 0.32	0.26	0.08	− 0.21	− 0.49	− 0.33	− 0.20	0.21	− 0.40*	− 0.14	− 0.03	0.13
AQLQ	0.25	− 0.15	− 0.01	0.40	0.47	0.40	0.24	0.11	0.38	0.40	0.35	− 0.06
PSQI	− 0.52*	0.64*	− 0.40	0.18	− 0.34	− 0.15	− 0.12	− 0.35	− 0.46	0.03	− 0.16	0.30
SIGH-A	− 0.21	0.32	0.00	− 0.08	− 0.11	− 0.33	− 0.18	− 0.14	− 0.38	0.15	-0.18	0.16
Leukocytes, × 10/9/l	0.19	− 0.25	0.28	− 0.08	0.52*	− 0.17	0.29	− 0.08	− 0.10	0.04	0.20	0.19
Eosinophils, × 10/9/l	0.27	− 0.30	0.22	− 0.21	0.31	− 0.13	0.25	0.35	− 0.03	0.14	− 0.07	− 0.12
Eosinophils, %	0.11	− 0.40	0.01	− 0.27	0.10	− 0.16	0.12	0.32	0.01	0.18	− 0.23	− 0.34
Neutrophils, × 10/9/l	0.02	− 0.03	0.21	0.08	0.44*	− 0.10	0.17	− 0.14	− 0.06	− 0.09	0.10	0.33
Neutrophils, %	− 0.08	0.00	0.18	0.25	0.30	0.10	0.17	− 0.26	0.23	0.23	− 0.03	0.25
Eosinophils in nasal smear, %	0.21	− 0.46	0.25	− 0.51*	0.31	− 0.19	0.14	0.38	− 0.05	− 0.33	− 0.03	0.12
Total IgE, kU/l	0.18	− 0.12	0.74**	0.25	0.35	0.23	− 0.09	− 0.17	− 0.16	− 0.00	0.33	0.20
Vitamin D, IU/ml	0.13	− 0.16	− 0.07	− 0.05	− 0.25	0.17	− 0.32	0.21	− 0.12	− 0.29	− 0.04	− 0.07
FEV1, l, mean ± SEM	0.08	− 0.16	− 0.03	0.33	− 0.14	0.40	0.56*	0.11	0.48	0.38	0.10	− 0.16

*p < 0.05, ^**^p < 0.01

Serum IL-22 negatively correlated with IL-22 in nasal lavage, whereas serum IFN-gamma positively correlated with IFN-gamma in nasal lavage. No correlations of other cytokines (IL-10, IL-13, IL-17, IL-33) were found between serum and nasal lavage. Serum IL-17 highly positively correlated with serum IL-33 as well as IL-17 and IL-33 in nasal lavage. Other significant positive correlations were found between serum IL-22 and serum IL-10, IL-17, IL-33, and IFN-gamma. All correlations are presented in Table 4.

**Table 4:** Correlation between cytokines in patients with allergic airway diseases

	Serum IL-22	IL-22 in nasal lavage	Serum IL-13	IL-13 in nasal lavage	Serum IL-10	IL-10 in nasal lavage	Serum IL-17	IL-17 in nasal lavage	Serum IL-33	IL-33 in nasal lavage	Serum IFN-gamma	IFN-gamma in nasal lavage
Serum IL-22	–	− 0.35**	0.35**	0.06	0.49**	0.28*	0.50**	− 0.05	0.53**	0.07	0.55**	0.19
IL-22 in nasal lavage	− 0.35**	–	− 0.08	0.10	− 0.08	− 0.06	− 0.01	0.16	− 0.06	0.17	− 0.24	− 0.12
Serum IL-13	0.35**	− 0.08	–	0.17	0.60**	0.12	0.21	0.13	0.30*	0.19	0.16	0.03
IL-13 in nasal lavage	0.06	0.10	0.17	–	0.23	0.27*	− 0.01	0.50**	0.15	0.49**	0.18	0.31*
Serum IL-10	0.49**	− 0.08	0.60**	0.23	–	0.20	0.30*	0.30	0.42**	0.34**	0.34**	0.13
IL-10 in nasal lavage	0.28*	− 0.06	0.12	0.27*	0.20	–	− 0.04	0.05	− 0.14	0.22	0.23	0.37**
Serum IL-17	0.50*	− 0.01	0.21	− 0.01	0.28*	− 0.04	–	− 0.01	0.72**	− 0.02	0.50**	0.18
IL-17 in nasal lavage	− 0.05	0.16	0.13	0.45**	0.25	0.05	− 0.01	–	0.12	0.90**	0.06	0.16
Serum IL-33	0.53**	− 0.06	0.30*	0.15	0.42**	− 0.14	0.72**	0.12	–	0.09	0.43**	0.15
IL-33 in nasal lavage	0.07	0.17	0.19	0.49**	0.34**	0.22	− 0.02	0.90**	0.09	–	0.12	0.27*
Serum IFN-gamma	0.55**	− 0.24	0.16	0.18	0.34**	0.23	0.50**	0.06	0.43**	0.12	–	0.37**
IFN-gamma in nasal lavage	0.19	− 0.12	0.03	0.31*	0.13	0.37**	0.18	0.16	0.15	0.27*	0.37**	–

^**^p < 0.01

^*^p < 0.05

### Risk for development of allergic airway diseases

Using logistic regression analysis we found that increase of serum IFN-gamma and age decreased the risk of AR for healthy individuals (Table 5).

**Table 5:** Risk for development of allergic airway diseases

	B	Sig	Exp (B)	95% Confidence Interval for Exp(B)	
				Lower bound	Upper bound
*Risk for development of AR for healthy individuals*					
Serum IFN-gamma	− 0.71	0.02	0.49	0.274	0.874
Age	− 0.08	0.04	0.92	0.851	0.996
*Risk for development of AA for patients with AR*					
Serum IL-22	0.14	0.02	1.15	1.024	1.299
Serum IL-10	− 0.27	0.01	0.07	0.009	0.504
IL-10 in nasal lavage	2.30	0.03	9.98	1.198	83.138
IL-33 in nasal lavage	0.57	0.05	1.77	1.011	3.091
Vitamin D	− 0.09	0.03	0.91	0.842	0.990

Risk for development of AA for patients with AR increased when levels of IL-22 in serum and IL-10 and IL-33 in nasal lavage increased. This risk decreased when serum IL-10 and vitamin D levels were higher.

## Discussion

Our study revealed that Th1, Th2 and Th17 produced cytokines, vitamin D and interaction between these biomarkers may be important in the pathogenesis of persistent allergic airway inflammation in patients sensitized to house dust mite, may increase the risk of development of AR and AA, and may be related with the course of disease. We investigated inflammatory biomarkers for patients with mild or moderate persistent allergic airway diseases because usually this type of research is conducted in severe forms of allergic diseases. However, the early diagnosis and apropos treatment is very important for prevention of severe forms of disease [41]. Moreover, AR and AA are associated with impaired quality of life even in patients with mild or moderate forms of disease [42, 43]. We found that persistent allergic airway diseases were associated with poor sleep quality. Our results are in agreement with other authors who found that AR and AA were associated with different sleep problems, such as significantly higher sleep quality scores, more frequent use of sleep medications, and abnormalities in polysomnography [44–46].

We have investigated local and systemic biomarkers and link between them. We found only one cytokine—IFN-gamma—which level positively correlated between nasal lavage and serum. IL-22 in serum negatively correlated with IL-22 in nasal lavage, whereas no correlation of other investigated cytokines was found between different compartments. This finding suggests that measurement of cytokines in serum may not always reflect the local level. This inadequacy could be explained by the severity of the diseases. For example, He et al. found that mild atopic dermatitis which pathogenesis is similar to allergic airway diseases showed high levels of Th2/Th22 cell activation in localized skin lesions and lacked the systemic inflammation [47]. All patients who were involved in our study also had mild or moderate form of AA and/ or AR.

IL-10 is known as anti-inflammatory cytokine [48]. We have found that serum IL-10 level was higher in patients with AR than in patients with both AR and AA suggesting that higher systemic concentration is found in milder form of allergic airway diseases where inflammation affects only upper airways. However, IL-10 level in nasal lavage increased risk for AA in patients with AR. Similarly, Xu et al. found that patients with a more severe endotype of nasal polyposis such as eosinophilic nasal polyposis, or patients with concomitant respiratory diseases such as asthma showed higher IL-10 expression levels in tissue samples [49]. This could be explained by the theory that increased levels of IL-10 can moderate the extent of apoptosis which is activated by inflammatory molecules [50].

One of the best investigated inflammatory biomarkers Th2 produced IL-13 in serum negatively correlated with quality of life in patients with AR and AA. These findings support theory of importance of Th 1 and Th2 ratio balance in pathogenesis of allergic airway diseases [2, 6, 14]. IL-13 is associated with eosinophilic lung inflammation, airway epithelial cell hypertrophy, goblet cell metaplasia, mucus hypersecretion, subepithelial fibrosis, and airway hyperresponsiveness [51, 52]. IL-13 promotes mast cells and B cell proliferation and induces class switching to IgE and IgG4, also promotes survival, activation, and recruitment of eosinophils and stimulates eosinophil trafficking from the peripheral blood to the site of inflammation by inducing the production of IL-5 and eosinophil chemokines such as eotaxins [53]. Clinical studies show increased serum, nasal lavage, sputum and bronchial lavage (BAL) IL-13 concentration in children and adults having atopic diseases including AA and AR [52, 54–59]. One recent study even revealed that IL-13 in children with wheezing can be considered as a possible predictor of asthma development [60].

We found that serum IL-17 positively correlated with total IgE, but IL-17 in nasal lavage negatively correlated with eosinophils in nasal smear in patients with AR and AA. The main function of Th17 cells is to clear extracellular and intracellular pathogens [61, 62] and to protect mucosal homeostasis and to enhance neutrophil response [61]. IL-17 is usually associated with development of chronic non-allergic airway inflammation and neutrophilic inflammation [14, 63, 64]. However, we did not find correlation between IL-17 and neutrophil count in peripheral blood. Studies provide evidences that IL-17 contributes to type-2 low asthma [65], and high level of IL-17 is found in patients with severe asthma [18–20]. However, recent studies suggest that neutrophilic inflammation could be involved in allergic airway diseases [66]. Moreover, a significant correlation has been found between serum IL-17 and eosinophil cationic protein [67] and hypothesis occurred that in Th2 dominant environment IL-17 can promote eosinophils survival and degranulation leading to chronic nasal inflammation in AR [21]. A number of studies have found high levels of serum IL-17 in patients with AR [67–69].

We found significant positive relation between IL-17 and IL-33 in serum and nasal lavage. The link between these cytokines were observed in other studies which investigated different disorders, such as sepsis and urticaria, which immunological mechanisms are similar to those in allergic airway diseases [70, 71]. Vocca et al. hypothesized that IL-33/ST2 axis could be involved in Th17 immune response during the progression of allergic airway disease, however the exact mechanism is not known [72]. According to the scientific literature, IL‐33 is associated with Th2 inflammation [9]. In agreement, we estimated that this cytokine in nasal lavage increased the risk for development of AA for patients with AR and had positive correlation with inflammatory cytokine IL-13.

Our study showed that serum IL-22 correlated with better lung function in patients with AR only and negatively related with PSQI score in patients with AR and AA. Moreover, serum IL-22 positively correlated with anti-inflammatory cytokine IL-10 in patients with allergic airway diseases. These data suggest about possible anti-inflammatory properties of this cytokine which are in agreement with experimental studies performed by other authors which showed that IL-22-positive mice had a reduced number of eosinophils and a lower level of IL-13 in bronchial lavage and mucus-producing cells in the airways after ovalbumin stimulation when compared with IL-22-negative mice [73, 74]. However, we estimated positive correlation between PSQI and IL-22 in nasal lavage. IL-22 in nasal lavage did not correlate with anti-inflammatory cytokines. Moreover, logistic regression showed that increase of serum IL-22 level increases the risk for development of AA for patients with AR. These results are in agreement with the results of majority of clinical studies which showed relation between IL-22 level and severity of AA and AR [75–77]. Zhao et al. revealed that the concentration of IL-22 in plasma was consistently increased with the severity of AA [78]. Our study provides quite controversial results regarding the role of IL-22, which can be explained by the theory that IL-22 could have an immunosuppressive effect only during the early stage of an immune response [79].

According to our study, IFN-gamma in nasal lavage was associated with better nasal symptoms score and quality of life in patients with AR only. Moreover, higher serum IFN-gamma decreased the risk for development of AR for healthy individuals. These findings support the theory that higher level of Th1 cytokines is associated with decreased risk of allergic diseases [80]. Experimental study showed that ablation of T cell–derived IL-10 increased the IFN-γ response to HDM, reducing IL-13 levels and airway eosinophilia without affecting IgE levels or airway hyperresponsiveness [81].

Although we did not find differences of vitamin D in studied groups, but logistic regression showed that vitamin D decreased the risk for development of AA for patients with AR. There is some evidence that low vitamin D level is associated with increased risk and more severe forms of allergic diseases [29, 30], although, the mechanisms are yet unclear. Experimental research showed that vitamin D suppressed the production of IgE and increased IL-10 production, suppressed mast cell activation, and reduced proinflammatory cytokine production [11]. However, we did not find correlations between vitamin D and these markers.

It is known that AR usually affects children and young people. The prevalence of seasonal AR is higher in children and adolescents, while perennial AR seems to be more common in adults [6]. Our study showed that older age decreases the risk for development of AR for healthy individuals. The similar results provided Cazzoleti et al. who showed AR decrease from 26.6% in 20–44 years age class to 15.6% in the 65–84 years age class [72].

## Conclusions

To sum up, novel cytokines such as IL-22, IL-17 and IL-33 may be involved in pathogenesis of persistent airway inflammation in patients sensitized to HDM, however their exact mechanisms and interrelationship still is not known. Th1, Th2 and Th17 produced cytokines and IL-33 correlate with symptoms score, lung function, eosinophil count and total IgE and may be biomarkers for prognosis and diagnosis of AR and AA in the future. However, more studies still need to be performed. Moreover, age and vitamin D level could be the risk factors for development of allergic airway diseases.

## Data Availability

The datasets used and/or analysed during the current study are available from the corresponding author on reasonable request.

## Abbreviations

AR: Allergic rhinitis
AA: Allergic asthma
Th: T lymphocyte helper
HDM: House dust mites
IL: Interleukin
IFN: Interferon
Ig: Immunoglobulin
TNSS: Total nasal symptom score
RQLQ: Rhinoconjunctivitis Quality of Life Questionnaire
ACT: Asthma control test
AQLQ: Asthma Quality of Life Questionnaire
PSQI: Pittsburgh Sleep Quality Index
SIGH-A: Structured Interview Guide for the Hamilton Anxiety Scale
FEV1: Forced expiratory volume in 1 s
FVC: Forced vital capacity
